# Using ‘smart regulation’ to tackle antimicrobial resistance in low-income and middle-income countries

**DOI:** 10.1136/bmjgh-2019-001864

**Published:** 2020-01-30

**Authors:** Gerard Porter, Jyoti Joshi, Lovleen Bhullar, Anita Kotwani, the Smart Regulation of Antibiotic Use in India: Understanding, Innovating and Improving Compliance Project Group, Nagendra Hegde, Anjana Sankhil Lamkang, Vishal Bansal, Ranjana Bharti, Lucy Kimbell, Meenakshi Gautham, Javier Guitian, Ana-Despina Tudor

**Affiliations:** 1 School of Law, The University of Edinburgh, Edinburgh, Midlothian, UK; 2 Center for Disease Dynamics Economics and Policy, New Delhi, Delhi, India; 3 Amity Institute of Public Health, Amity University, Uttar Pradesh, India, India; 4 Department of Pharmacology, V. P. Chest Institute, University of Delhi, Delhi, India

**Keywords:** health policy, public health

**Summary box**

- Low-income and middle-income countries are aligning their National Action Plans on antimicrobial resistance with WHO’s 2015 Global Action Plan.
- Regulation is a key tool for operationalising national standards aimed at optimising the use of antimicrobial medicines.
- On its own, the traditional command-and-control approach to regulation is poorly suited to this challenge.
- ‘Smart regulation’ can be used to supplement, fine-tune and improve on more traditional regulatory approaches.

## Introduction

Antimicrobial resistance (AMR) contributes to over 700 000 annual deaths globally. According to the O’Neill report, an estimated 10 million lives a year and a cumulative US$100 trillion of economic output will be at risk by 2050 if we do not put in place proactive solutions now. Tackling AMR is a multifaceted task that requires a One Health approach encompassing human, animal and environmental health as suggested in the WHO’s 2015 Global Action Plan (GAP) on AMR. Countries around the world have aligned their National Action Plans (NAPs) on AMR with this international guidance.

One of the important links for various activities for AMR containment is the appropriate use of antibiotics to reduce selection pressure on microbes. In low-income and middle-income countries (LMICs), however, antibiotic consumption rates have been converging towards (and in some countries surpassing) levels typically observed in high-income countries.^1^ Further, a higher burden of infectious diseases and restricted access to new antibiotics suggest a higher burden of AMR in LMICs than in high-income countries.^2^ Finally, there is rampant misuse or overuse of antibiotics in humans^3 4^ and food animals as well as their exposure in the environment.^5 6^ While the nature of pathways for the emergence and transmission of AMR and the functionality of the regulatory system across various sectors might differ among LMICs, they all face this core public health challenge. There is therefore a pressing need for effective design and implementation of activities for NAP-AMR in LMICs. This paper contributes to the literature on AMR regulation by discussing the approach known as ‘smart regulation’ and outlining how it may be used to supplement, fine-tune and improve upon more traditional regulatory approaches for optimum use of antibiotics in One Health sectors.

## AMR and the traditional approach to regulation

WHO’s GAP identified regulation as a key tool for ensuring the operationalisation of national standards to optimise the use of antimicrobial medicines in human and animal health. More recently, the United Nations Inter-Agency Coordination Group on AMR also recommended the development, maintenance, strengthening and implementation of AMR regulation for human, animal and environmental health.

Traditionally, the term ‘regulation’ refers to the top-down command-and-control model of regulation, which imposes standards and sanctions in case of non-compliance with the standards.^7^ This traditional model is a key feature of the regulatory landscape for some of the plausible pathways of AMR in a number of LMICs. For instance, laws prohibit over-the-counter (OTC) sales of antibiotics by pharmacists without a prescription from a registered medical practitioner, empower drug inspectors to monitor compliance and punish violation with a fine or imprisonment or both.^8^ For a variety of reasons, however, the enforcement of such laws in LMICs may be patchy or non-existent.^9^ For other pathways, such as the disposal of antibiotic residues in effluent from antibiotic manufacturing, laws are in a nascent stage or non-existent.

When viewed from a traditional regulatory perspective, legislators can solve the problem of gaps in the law by formulating appropriate regulatory standards. Once appropriate laws are on the statute books, non-compliance can be addressed through measures including greater investment in regulatory infrastructure, increasing the numbers of trained regulators and resolving instances of confusion, overlap or fragmentation of responsibilities among the array of regulatory bodies charged with enforcing the laws. Although these ways of bolstering the traditional regulatory approach are indeed valuable and necessary, they may not be sufficient to address the complex challenges at hand.

One problem with the traditional regulatory perspective is that it envisages a top-down relationship between the regulator and the regulated entity. It often does not allow space for meaningful engagement between the two, or with the range of other actors who directly or indirectly influence and/or are affected by regulation. This is problematic, particularly in LMICs, as regulations may be drafted without fully appreciating situated interests and how socioeconomic and structural factors underpin behaviour. The regulatory standards that emerge may therefore fail to secure widespread support. Private stakeholders may resist or actively contest regulatory policies if these are perceived as threatening their interests.^10^ Recent WHO^11^ and Food and Agriculture Organization (FAO)^12^ guidance highlights the importance of multisectoral coordination and engaging diverse stakeholders as an aspect of successful implementation of AMR NAPs. There is scope for further discussion, however, around optimum methodologies for achieving these goals. Furthermore, although existing recommendations for regulatory reform in some LMICs already envisage multistakeholder engagement in some sectors, this typically will not be advocated at an upstream stage when stakeholders might have meaningful input into the regulatory design process. ‘Smart regulation’ is worthy of consideration in relation to these and other points.

## ‘Smart regulation’ of AMR

Gunningham *et al* coined the term ‘smart regulation’ ‘to overcome the inefficiencies of traditional regulation on the one hand, and the pitfalls of deregulation on the other’.^13^ According to them, smart regulation describes a form of ‘regulatory pluralism’ that embraces much more ‘flexible, imaginative and innovative forms of social control’ than conventional regulation. The authors asserted that not only could smart regulation be effective in delivering policy objectives; it can also increase efficiency by doing so at least cost to the government, business and the community. Furthermore, it was developed as a useful way to approach regulating complex areas that involve multiple stakeholders with converging and diverging interests.

Designing smart regulation involves two stages. First, the policymakers should identify the desired policy goal(s) and the trade-offs necessary to achieve it, the unique characteristics of the problem, the range of potential actors and instruments and opportunities for consultation and public participation. Then, they should apply five enabling core regulatory design principles sequentially to arrive at solutions (box 1).

Box 1 **Core regulatory design principles**

- Incorporate a broad range of complementary instruments and actors.
- Prefer least interventionist (prescriptive or coercive) but viable instruments.
- Create a ‘regulatory enforcement pyramid’ that comprises three faces representing first parties (government), second parties (business) and third parties (commercial eg, financial markets and insurers and non-commercial, eg, non-governmental organisations and other groups) and set out progressively serious penalties for non-compliance across several instruments and different faces.
- Empower second and third parties to act as surrogate regulators.
- Optimise the opportunity for win-win outcomes.

Gunningham *et al* originally developed smart regulation in the context of environmental regulation, but they recognised that the implications of their research could extend to other domains. Subsequently, scholars have considered the application of smart regulation to other fields, including e-waste,^14^ shipping^15^ and health regulation.^16^ Policymakers have already applied the concept across different sectors, including the environment, agriculture, food safety and health in Canada^17^ and the European Union.^18^ Although most of these developments have taken place in western countries, we suggest that smart regulation offers a useful framework to address the challenge of AMR in LMICs.

To illustrate, we discuss how the above five core regulatory design principles of the smart regulation approach might be applied in practice, using OTC sales of antibiotics without a valid prescription (which is rampant in LMICs) as an example. For the first principle, a broad range of instruments and actors could be involved in the design and implementation of regulation. Actors could include government/regulators, pharmacists, associations of pharmacists, consumers, representatives of consumers, non-governmental organisations (NGOs), area resident welfare associations, community/religious leaders, local government bodies, pharmaceutical companies and their sales representatives, importers, wholesalers and distributors of antibiotics. In terms of instruments, traditional regulation, voluntary codes of conduct, awareness/information campaigns and the use of technology could all be considered. For the second design principle, the least interventionist but viable instrument could take the form of education and awareness instead of immediate imposition of a penalty. For the third principle, the regulatory enforcement pyramid could comprise the government/regulator (first face), pharmacists and associations of pharmacists (second face) and consumers, representatives of consumers, NGOs, area resident welfare associations, community/religious leaders, local government bodies, pharmaceutical companies and their sales representatives, importers and wholesalers and distributors of antibiotics (third face). Progressively serious penalties for non-compliance could be set out across several instruments and different faces. This could take the form of: education and awareness, then a warning, followed by a second warning and a nominal fine, then temporary closure of pharmacy and a higher fine, then ultimately imprisonment. For the fourth principle, surrogate regulators could include the actors represented in the second and third faces, harnessing their ability to implement forms of regulation including self-regulation. Fifth, all the above-mentioned actors could work together to explore and optimise opportunities for win-win opportunities. In this case, this could mean attempting to satisfy public health interests (ie, that antibiotics are not overused or misused) alongside ensuring that the economic interests of pharmacists as business owners and members of the community are also supported.

## Conclusion

Some existing regulatory arrangements and policy proposals^11 12^ for implementing NAPs on AMR in LMICs already advocate moving beyond a traditional regulatory approach by emphasising the importance of multisectoral coordination and stakeholder engagement. To these suggestions, smart regulation adds further useful elements, including a stronger emphasis on involving stakeholders in the design of regulatory standards (ie, not just in the implementation of standards they have been unable to shape) across all relevant policy areas, the deployment of a broader range of regulatory tools and the goal of developing win-win regulatory options whenever possible.

Smart regulation utilises the existing institutional, legal and governance framework, including the traditional command-and-control approach, but also expands options for regulators. It could encourage behaviour change, incentivise regulatory compliance and ensure the most efficient and effective application of the resources of different actors as well as greater acceptance and smoother implementation of regulatory instruments. By taking into account the consequences of regulation on different actors from the outset, it might also be possible to avoid or mitigate problems arising from the unintended consequences of AMR regulation. This would also represent an advance from the perspective of equity, which is critical in LMICs.

Of course, the successful operationalisation of smart regulation of AMR would be contingent, among other factors, on sequential application of all of the core regulatory design principles, including ensuring the complementarity of actors and instruments and viability of instruments, as well as effective monitoring mechanisms to avoid unintended consequences, in addition to avoiding regulatory capture. With awareness of these issues, LMICs can harness the advantages offered by smart regulation to develop more efficient, workable and effective regulatory frameworks for tackling AMR.
